# Sex versus Gender Role Endorsement Associations With White Matter Microstructure in South African Adults: A Diffusion Tensor Imaging Study

**DOI:** 10.1002/brb3.71340

**Published:** 2026-03-31

**Authors:** Hilmar Klaus Luckhoff, Retha Smit, Lebogang Phahladira, Sanja Kilian, Robin Emsley, Laila Asmal

**Affiliations:** ^1^ Department of Psychiatry, Faculty of Medicine and Health Sciences Stellenbosch University Cape Town South Africa

**Keywords:** diffusion tensor imaging, gender, sex, white matter

## Abstract

**Background:**

Brain structure in transgender individuals is often shifted away from their natal sex toward their preferred gender identity. However, little is known about the relationship between gender identity and white matter microstructure in the general population.

**Methods:**

We investigated the associations of biological sex versus gender role endorsement with white matter microstructure in healthy South African adults (*n* = 88; 46 males, 42 females) meticulously screened for mental illness, medical comorbidities, intracranial pathologies, significant head trauma, and substance use. Gender role endorsement was assessed as a dimensional measure across a continuum using the Bem Sex Role Inventory. Diffusion tensor imaging was used to calculate fractional anisotropy (FA) for selected fronto‐limbic white matter tracts of interest.

**Results:**

We observed a significant interaction effect between biological sex and gender role endorsement across multiple white matter tracts, including the trunk of the corpus callosum, left corticospinal tract, left superior longitudinal fasciculus, right cingulum bundle, right inferior fronto‐occipital fasciculus, and left uncinate fasciculus. Post‐hoc tests revealed that higher feminine gender role endorsement was associated with lower FA in these tracts among males, but not females.

**Conclusions:**

Gender nonconformity in males is linked to a “*sex‐reversed*” pattern of white matter microstructure in healthy adults recruited from a general population setting. Future longitudinal studies are needed to investigate how sex, gender identity, genetics, sex hormones, and environmental exposures interact to shape white matter development across the lifespan.

## Graphical abstract

Gender nonconformity in males is associated with sex‐reversed white matter microstructural patterns across multiple fronto‐limbic tracts, while no such effects are observed in females. These findings highlight sex‐specific links between gender role endorsement and brain structure in the general population.

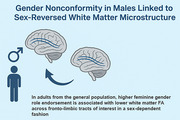

## 1 Introduction

Gender is considered a “*core facet of the self*” (Jacobson and Joel 2019). The terms “*sex*” and “*gender*” are often conflated in academic and public discourse. “*Sex*” traditionally refers to biological differences between males and females, including chromosomal complement, sex hormone levels, and reproductive anatomy. These traits, however, exist along a spectrum, and not all individuals fit neatly into male or female categories—for example, intersex individuals may present atypical combinations. In contrast, “*gender*” is a social construct encompassing culturally defined behaviors, beliefs, and expressions associated with masculinity and femininity. Individuals often display a continuum of masculine and feminine traits, rather than conforming to rigid, mutually exclusive categories (Coen and Bannister 2012).

The formation and consolidation of gender identity is influenced by cumulative events during critical developmental periods when the brain is differentiating under the influence of sex hormones, genes, and maternal factors (Rosselli 2018). The clinical expression of gender nonconforming identities is linked to pre‐ and initial postnatal sex hormone exposure, leading to less prominent sexual differentiation in the brain (Garcia‐Falgueras and Swaab 2010). Trans‐identities might thus be rooted in a mismatch between sex‐specific patterns of brain versus genital development (Mueller et al., 2017). Whole‐brain tract network analysis has indeed shown that male‐to‐female (MtF) transgender individuals have increased inter‐hemispheric subcortical‐limbic connectivities compared to cisgender males, while female‐to‐male (FtM) transgender individuals had decreased intra‐hemispheric connectivities compared to cisgender females (Hahn et al., 2015).

Neuroimaging research has allowed researchers novel insights into the neurobiological substrates of gender identity, showing that brain structure in gender non‐conforming individuals is shifted away from their natal sex toward their preferred gender identity (Luders et al. 2012; Zubiaurre‐Elorza et al. 2013). Diffusion tensor imaging (DTI) studies indicate that transgender individuals show “*sex‐reversed*” patterns of white matter microstructural connectivity (Kranz et al. 2014; Burke et al. 2017). In other words, the white matter microstructural properties of transgender individuals often resemble those typically observed in cisgender individuals of their identified gender rather than their natal sex. For example, DTI studies have found that MtF transgender individuals show higher fractional anisotropy (FA) in fronto‐limbic tracts—including the corpus callosum (forceps minor), superior longitudinal fasciculus (SLF), corticospinal tract (CST), cingulum bundle, and inferior fronto‐occipital fasciculus (IFOF)—patterns more characteristic of cisgender females than cisgender males (Rametti et al., 2011a,b, 2014; van Heesewijk et al., 2022).

These frontolimbic white matter tracts subserve self‐referential thinking, bodily self‐processing, interhemispheric integration, emotional salience attribution, and social‐cognitive operations implicated in gender identity formation (Burke et al. 2017; van Heesewijk et al., 2022). Several other fronto‐limbic tracts, such as the fornix and uncinate fasciculus (UF), exhibit sex‐based variation in white matter structure, in addition to being implicated in the integration of self‐concept, emotion regulation, and social‐affective evaluation, processes that may be particularly relevant to the development and consolidation of gender identity (Von Der Heide et al. 2013; Zuurbier et al. 2013; Coad et al. 2020; Herlin et al. 2024; Torgerson et al. 2024). The proposed associations between gender identity and white matter integrity likely emerge through multifactorial developmental pathways, wherein prenatal and pubertal sex hormone exposure exerts organizational effects on fronto‐limbic circuits mediating emotion regulation and self‐concept (Kranz et al. 2017; van Hemmen et al. 2017). Socially reinforced gendered behaviors may also drive experience‐dependent plasticity through repeated engagement of cognitive, affective, and social processing networks (Arraiza Zabalegui 2024). However, the precise mechanisms linking these bio‐psycho‐social factors to specific white matter phenotypes remain incompletely characterized.

Using a male–female distinction and classifying gender as binary in neuroimaging research has so far provided inconsistent insight into the relationship between gendered behaviors and brain structure (Eliot 2025). Earlier DTI studies are difficult to interpret partly because gender was treated dichotomously rather than as a continuum, obscuring meaningful inter‐individual variability (Rauch and Eliot 2022; Joel 2021). This limitation is increasingly recognized as both conceptual and ethical: binary frameworks can risk pathologizing gender nonconforming identities and reinforcing stigma by framing them as deviations from a normative standard (Eliot 2025; Edmiston and Juster 2022). Since gender is multidimensional, biological attributes cannot fully capture social and cultural identities (Levin et al., 2023). Even though biological sex is also multidimensional and often aligns with gender expression, it does not encompass all social, cultural, and psychological dimensions of gender.

The historic reliance on binary sex classifications in neuroimaging research has constrained our understanding of how inter‐individual variation in gendered behaviors is related to brain structure, independent of biological sex (Rauch and Eliot 2022; Eliot 2025). It remains unclear whether observed white matter differences reflect biological sex or psychosocial dimensions of gender. While some studies have used non‐binary measures, including gender inventories and brain‐derived metrics, these have yet to yield definitive insights (Rauch and Eliot 2022). Reconceptualizing gender as a continuous spectrum may offer a more nuanced framework for understanding brain structure, yet research linking psychosocial and nonbinary dimensions to white matter microstructure remains scarce (Eliot 2025). Limited evidence, however, does indicate that gender, independent of sex, is associated with inter‐individual variations in brain structure (Wood et al. 2008; Pletzer 2019). For example, higher scores on a masculine–feminine continuum correlate with larger frontal white matter volumes in children and adolescents (Belfi et al. 2014), while gender identity correlates with fronto‐limbic connectivity (Rauch and Eliot 2022). The consideration of both gender role endorsement and biological sex is essential for understanding social and neurobiological influences on brain structure.

In the present study, we addressed these empirical gaps by examining the associations of biological sex and gender role endorsement with white matter microstructure in a healthy adult sample carefully screened for medical and psychiatric confounds. This design allowed us to disentangle their relative contributions to white matter organization and offered novel insights into how socially endorsed gender roles may shape brain structure beyond biological sex. First, we compared white matter FA for both global and regions‐of‐interest (ROIs) measures between males and females. Second, we explored the effects of biological sex and gender continuum scores as well as their interactions on white matter FA. Third, we examined anticipated sex‐specific associations of individual masculine and feminine gender constructs with white matter FA in males compared to females. We hypothesized that (1) males would have higher white matter FA than females (Hsu et al. 2008; Kanaan et al. 2012; Burke et al. 2017) and (2) gender role endorsement patterns not aligned with biological sex would be associated with “*sex‐reversed*” white matter microstructural properties (Rametti et al. 2011a, b; Kranz et al. 2014; Burke et al. 2017; van Heesewijk et al. 2022).

## 2 Methodology

### 2.1 Study Design

This was a cross‐sectional study that included healthy adult participants (*n* = 88) previously enrolled as controls in two parent studies conducted in Cape Town, located in the Western Cape province of South Africa. The first explored multidimensional aspects of treatment outcome, including brain structural correlates in patients with schizophrenia compared to healthy controls (2007–2011) (EONCKS study (N06/08/148)), while the second (2013–2017) explored the underpinnings of metabolic syndrome and serious mental illness, including the relevance of brain structural differences between cases and controls (SHARED ROOTS study (N13/08/115)).

### 2.2 Participants

We included healthy control participants recruited through personal contacts of the families of patients included in the parent studies, as well as advertisements placed in community centers in the same catchment areas. Biological sex was defined at the time of interview in all participants based on self‐report as male (*n* = 46) or female (*n* = 42). Exclusion criteria were (1) a current DSM‐IV‐TR axis I diagnosis, (2) a serious general medical condition that would have impacted the DTI assessment, (3) substance use or dependence, (4) significant head trauma, (5) intracranial pathologies, and (6) a positive urine toxicology result for cannabis, methamphetamine, or methaqualone. For the neuroimaging protocol, participants were excluded if they had a cardiac pacemaker, metal prosthesis or pin(s), clips on blood vessels, inner ear prosthesis, an infusion pump, a metal intra‐uterine contraceptive device, claustrophobia, or current pregnancy or were too large to fit into the head scanner.

### 2.3 Gender Role Endorsement

Here, we aimed to clarify how socially embedded gender dimensions relate to inter‐individual variation in white matter microstructure (FA). We therefore decided to focus on gender role endorsement in particular as a reflection of how individuals understand and engage with social expectations around their gender. In contrast, gender identity shows a less direct link with behavioral expectations and the social positioning of gender. This makes role endorsement a more ideal proxy marker for the social processes that could shape brain structure.

In this study, gender role endorsement was measured using the Bem Sex Role Inventory (BSRI) (Bem, 1981), a widely used self‐report scale assessing masculine and feminine traits on a 7‐point continuum. The BSRI provides separate masculinity and femininity scores, conceptualized as distinct constructs, rather than opposite ends of a single binary. Extensive evidence supports the reliability and internal consistency of the original and short‐form BSRI (Cronbach's α ≈ 0.70–0.90), as well as their construct validity and stable factor structures, when measuring gender role endorsement in cis‐ and transgender individuals across diverse populations and cultural settings (Choi and Fuqua 2003; Choi et al. 2009). In the present sample (n = 88), internal consistency as indexed by Cronbach's alpha was good for both the BSRI masculinity (α = 0.83) and femininity (α = 0.80) subscale domains. The BSRI has also been translated into multiple languages (Hoffman and Borders 2001) and used in the South African population (Geldenhuys and Bosch 2020). BSRI scores were standardized, and no missing data were present.

Similar to Belfi et al. (2014) as well as our prior neuroimaging research (Luckhoff et al., 2024), we calculated a continuum score as a global measure of gender by subtracting the masculinity score from the femininity score (F—M). Higher continuum scores thus indicated a more feminized gender profile in relation to biological sex, while lower continuum scores indicated a more masculinized gender profile. In the event where a significant effect was observed for gender continuum scores in our primary analyses, masculinity and femininity scores were examined separately in relation to white matter microstructure in our secondary analyses.

### 2.4 Neuroimaging

For all participants included in our study, high‐resolution diffusion‐weighted images (DWI) were acquired on an Allegra MRI scanner (Erlangen, Germany) with the following parameters: field of view = 220 mm, spatial resolution = 1.8 mm × 1.8 mm × 1.8 mm^3^, repetition time = 8800 ms, echo time = 88 ms, 65 slices, slide gap = 0, with two‐fold GRAPPA acceleration. The gradients were applied in 30 directions with *b*  =  1000 s/ms^2^ and a single unweighted volume (*b*  =  0/mm^2^) was also acquired. This sequence was repeated three times. The DWI was pre‐processed using the Functional MRI of the Brain (FMRIB) Software Library (FSL) version 4.1.8. We used the standardized TBSS pipeline (in FSL) with tract‐specific ROIs defined using the JHU White‐Matter Tractography Atlas to ensure consistent between‐participant measurements. Raw DTI data were corrected for eddy current distortions and head motion, and the images were imported into MATLAB. The three acquisitions were co‐registered by using the first *b*  =  0 mm/s^2^ as the reference image. During standard quality control procedures, we screened for potential outliers using *Z*‐values derived from the 25th and 75th percentiles of the FA distribution, with a predefined threshold of >3 standard deviations (SD) used to meet criteria for exclusion. No observations, however, met this criterion.

#### 2.4.1 Neuroimaging Outcomes of Interest

##### 2.4.1.1 Global White Matter FA Measure

We calculated an overall measure of white matter microstructure by averaging mean FA values across selected white matter tract ROIs for each participant. This unweighted composite reflected overall white matter microstructure, assuming an equal contribution for each tract to the global FA measure of interest.

##### 2.4.1.2 Loco‐Regional White Matter FA Measures

For the present research, we selected the following white matter tract ROIs (*n* = 14), based on their associations with gender identity, role endorsement, and self‐concept: the corpus callosum (trunk, genu, splenium), fornix, left and right SLF, left and right CST, left and right cingulum bundle, left and right IFOF, and left and right UF.

### 2.5 Statistical Testing

All statistical analyses were performed in R (version 3.4.5). RStudio (version 1.1.456) was used as the integrated development environment for code execution and reproducibility.

#### 2.5.1 Sample Characteristics

Categorical data were described as counts and percentages (%) and compared between males and females using the Chi‐square test. Continuous numerical data that fit normal distribution assumptions were described by the mean and standard deviation (SD) and compared between males and females using a Student's *t*‐test. Non‐normal data were described by the median and interquartile range (IQR) and compared between males and females using the Wilcoxon rank‐sum test. For continuous variables analyzed with parametric tests, Winsorization was applied in the presence of outliers, replacing values beyond the 1st and 99th percentiles with the nearest non‐extreme values to reduce their influence. Winsorization was not applied to variables analyzed with non‐parametric tests.

#### 2.5.2 Sex Differences in White Matter FA

Individual ANCOVA models were constructed to compare white matter FA between males and females, adjusting for age, highest level of education (HLOE), and total intracranial volume (TIV).

#### 2.5.3 Sex versus Gender Continuum Score Effects on Global White Matter FA

We constructed a hierarchical linear regression model to explore the independent effects of biological sex (block I) and gender continuum scores (block II) as well as their interactive effects (block III) on global white matter FA, adjusting for age, HLOE, and TIV. To assist with interpretation of our findings, post‐hoc partial Pearson correlations were run to examine the linear relationships between gender continuum scores and global white matter FA in males compared to females, adjusting for age, HLOE, and TIV.

#### 2.5.4 Sex versus Gender Continuum Score Effects on Loco‐regional White Matter FA

A multivariable hierarchical multiple linear regression model was constructed to examine the effects of biological sex (block I) and gender continuum scores (block II) as well as their interactions (block III) across the white matter tract ROIs, adjusting for age, HLOE, and TIV. Individual hierarchical regressions modeling sex and gender effects on white matter FA were then constructed based on the outputs from the multivariate tests. To assess the influence of individual observations on the final hierarchical regression models, leverage values and Cook's distance were examined. No cases exceeded conventional thresholds for influence (Cook's D > 4/[n − k − 1]), suggesting that the results were not unduly driven by any single participant. In order to control for multiple comparisons across the a‐priori ROI set (14 tracts), we applied the Benjamini–Hochberg false discovery rate (BH‐FDR) procedure (target *q* = 0.05) (Benjamini and Hochberg 1995) to the tract‐level tests for our primary predictors within the hierarchical regression models, with significance determined based on whether each test passed the BH‐FDR threshold derived from the ordered set.

#### 2.5.5 Sex versus Construct‐specific Gender Associations With White Matter FA

We used post‐hoc partial Pearson's correlations to explore possible sex‐specific associations between gender scores and white matter microstructure in males compared to females, adjusting for age, HLOE, and TIV. Given their exploratory nature, we did not correct for multiple comparisons in our post‐hoc tests.

### 2.6 Power Calculation

A post‐hoc power analysis was conducted following enrollment and data quality control using G*Power (v3.1.9.7) (Faul et al. 2009) to evaluate whether the achieved sample size was sufficient for the primary regression analyses. The analysis specified F‐tests for linear multiple regression (fixed model, *R* ^2^ increase), with two predictors of interest (biological sex and gender continuum scores) and three covariates (age, HLOE, and TIV). Input parameters were an effect size of *f* ^2^ = 0.13 (small), α error probability = 0.05, and target power (1–β) = 0.81, yielding a minimum required sample size of *N* = 80. The final dataset comprised N = 88 participants with complete tract‐level diffusion data following quality control, exceeding the minimum required sample size and indicating adequate sensitivity to detect effects of the specified magnitude in the primary regression models. This power analysis pertains specifically to the primary predictors and does not account for multiple testing across the 14 tract‐level outcomes; BH–FDR correction was applied in the primary analyses. Post‐hoc partial Pearson's correlations were considered exploratory and interpreted with caution, as they were not independently powered. The power analysis is reported to ensure transparency regarding the achieved sample size and its adequacy for addressing the primary research objectives.

## 3 Results

### 3.1 Sample Characteristics

**FIGURE 1:**
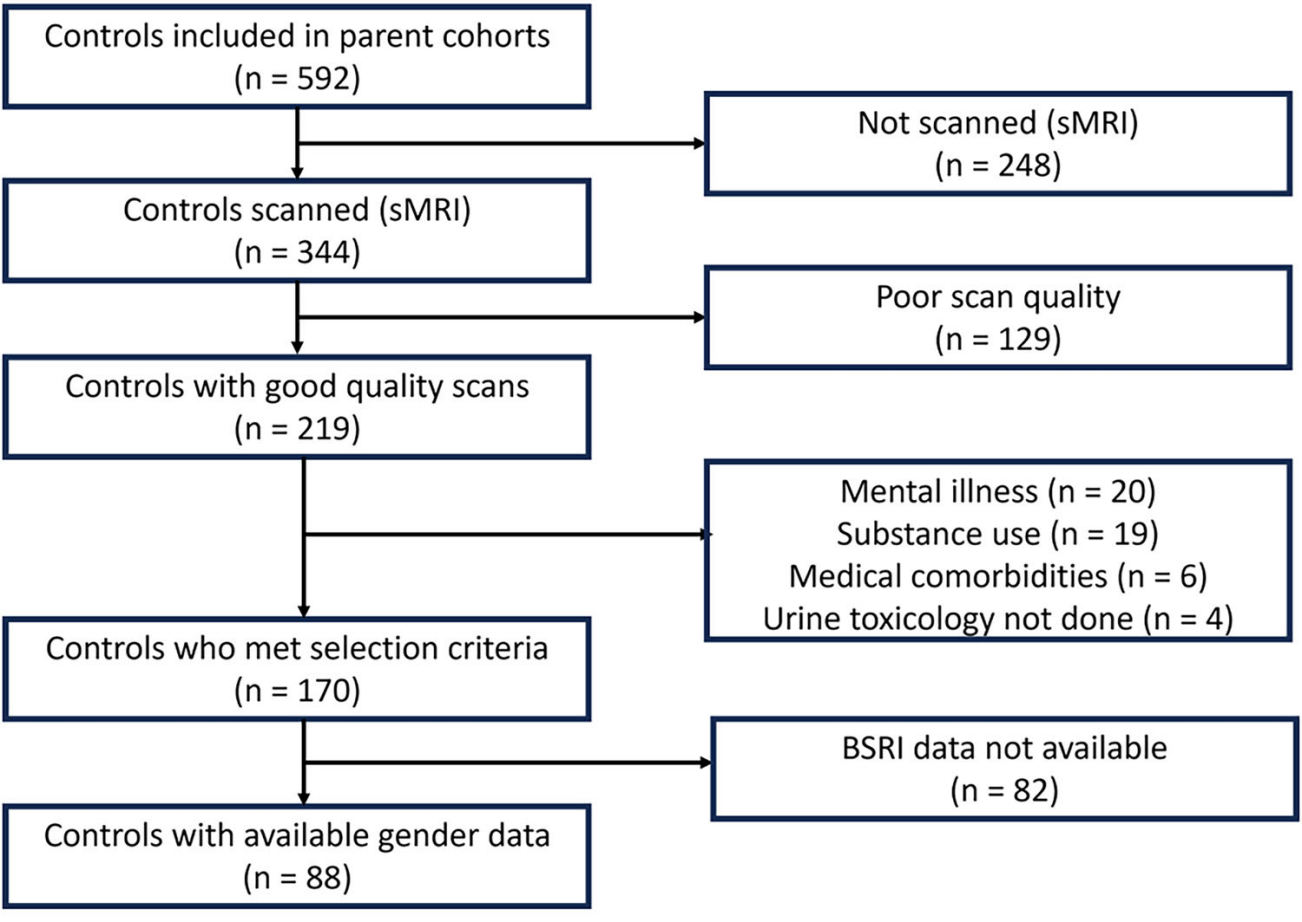
Selection criteria used to determine eligible participants. Abbreviations: BSRI, Bem Sex Role Inventory; sMRI, structural magnetic resonance imaging.

The procedures used to identify eligible participants are summarized in Figure 1. The final sample (*n* = 88) included 46 males and 42 females, who were balanced for age and HLOE. Gender role endorsement scores were similar in males compared to females (Supplementary Table S1).

### 3.2 Sex Differences in White Matter Microstructure

Individual ANCOVAs were used to compare (1) global and (2) loco‐regional white matter FA between males and females, adjusting for age, HLOE, and TIV (Table 1). The results indicated that males had higher global white matter FA than females. We also found that males had higher white matter FA for the trunk of the corpus callosum, left SLF, and left CST than females, correcting for multiple comparisons (Table 1).

**TABLE 1:** ANCOVA outputs showing comparisons of individual white matter tract fractional anisotropy (FA) compared between males and females, adjusting for age, highest level of education, and total intracranial volume. Data are presented as the mean along with standard deviation.

Brain region	Males (*n* = 46)	Females (*n* = 42)	*F*‐statistic (1, 86)	*p*‐value
**Global FA**	**0.45 (0.070)**	**0.41 (0.057)**	**8.70**	0.004*
**Loco‐regional white matter tracts of interest**				
**Corpus callosum trunk**	**0.44 (0.070)**	**0.40 (0.062)**	**8.09**	0.005*
Genu	0.44 (0.04)	0.43 (0.04)	1.37	0.77
Splenium	0.48 (0.05)	0.48 (0.03)	0.10	0.76
Fornix	0.46 (0.04)	0.45 (0.04)	0.08	0.78
**Left CST**	**0.59 (0.056)**	**0.56 (0.040)**	**8.47**	0.005*
Right CST	0.57 (0.11)	0.56 (0.11)	0.18	0.67
**Left SLF**	**0.35 (0.067)**	**0.31 (0.063)**	**8.35**	0.005*
Right SLF	0.40 (0.14)	0.39 (0.13)	0.12	0.73
Left cingulum bundle	0.42 (0.13)	0.41 (0.12)	0.14	0.83
Right cingulum bundle	0.43 (0.13)	0.40 (0.06)	1.95	0.08
Left IFOF	0.66 (0.15)	0.65 (0.15)	0.09	0.78
Right IFOF	0.28 (0.12)	0.28 (0.11)	0.92	0.34
Left UF	0.47 (0.07)	0.46 (0.05)	0.60	0.44
Right UF	0.35 (0.05)	0.35 (0.04)	0.15	0.75

*indicates statistical significance following correction for multiple testing (adjusted α = 0.005).

Abbreviations: CST, corticospinal tract; FA, fractional anisotropy; IFOF, inferior fronto‐occipital fasciculus; ROIs, regions‐of‐interest; SLF, superior longitudinal fasciculus; TIV, total intracranial volume; UF, uncinate fasciculus.

### 3.3 Sex versus Gender Effects on Global White Matter FA

**FIGURE 2:**
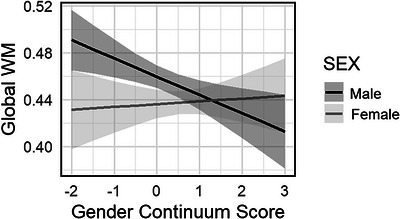
A predicted value plot showing sex‐specific associations between the gender continuum score and global white matter (WM) fractional anisotropy. The results are displayed separately for males (*n* = 46) and females (*n* = 42), with predicted values derived from the regression model.

We found a significant interaction effect between biological sex and gender continuum scores as predictors of global white matter FA, adjusting for age, HLOE, and TIV (Supplementary Table S2). Figure 2 shows predicted global white matter FA values from the hierarchical linear regression model, illustrating the sex × gender continuum score interaction, with separate regression lines for males and females. Post‐hoc partial Pearson's correlation testing revealed that there was a significant association between higher gender continuum scores and lower global white matter FA in males, adjusting for age, sex, and TIV (*r* = −0.37, *p* = 0.010). In contrast, there was a non‐significant positive correlation between gender continuum scores and global white matter FA in females, adjusting for the same covariates (*r* = 0.06, *p* = 0.689).

### 3.4 Sex versus Gender Effects on FA Measures for Fronto‐limbic White Matter ROIs

**FIGURE 3:**
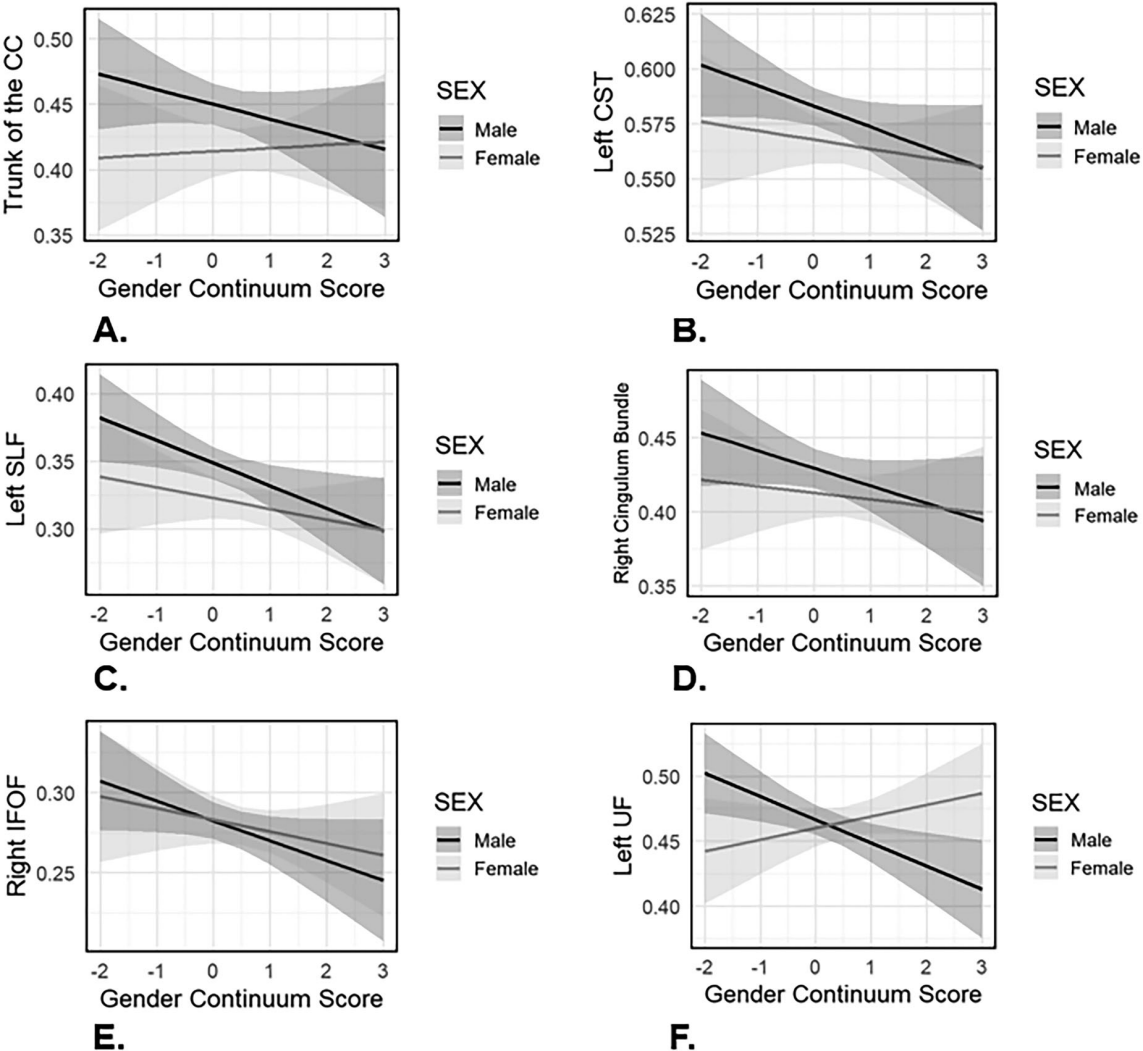
Predicted value plots illustrating sex‐specific associations between the gender continuum score and white matter fractional anisotropy (FA), shown separately for males (*n* = 46) and females (*n* = 42) for the: (A) Trunk of the corpus callosum (CC); (B) Left corticospinal tract (CST); (C) Left superior longitudinal fasciculus (SLF); (D) Right cingulum bundle; (E) Right inferior fronto‐occipital fasciculus (IFOF); and (F) Left uncinate fasciculus (UF). Predicted values are derived from the regression models examining sex × gender continuum score interactions.

Our results revealed a significant interaction effect between biological sex and gender continuum scores across the white matter tract ROIs (Pillai's Trace = 0.14, *F* (1,84) = 2.66, *p* = 0.021, partial *η* ^2^ = 0.13), adjusting for age, HLOE, and TIV (Table 2). Individual hierarchical regression models showed significant sex and gender interaction effects for the left CST, left SLF, corpus callosum trunk, right cingulum bundle, right IFOF, and left UF (Table 3). These associations all survived correction for multiple comparisons. Figure 3A‐F shows separate predicted value plots for white matter FA illustrating the significant sex × gender continuum score interaction effects for these ROIs. Individual hierarchical linear regressions did not, however, reveal any significant effects of biological sex, gender continuum scores, or their interaction for the genu or splenium of the corpus callosum, fornix, right SLF, right CST, left cingulum bundle, left IFOF, or right UF (Supplementary Table S3).

**TABLE 2:** Outputs from our multivariable hierarchical multiple linear regression model showing the effects of biological sex (block I) and gender continuum scores (block II) as well as their interactions (block III) on FA values across the white matter tracts of interest, adjusting for age, highest level of education, and total intracranial volume.

Predictor		*F*‐value	df	*p*‐value
Block I				
Sex		1.89	1, 84	0.036*
Block II				
Sex		1.93	1, 84	0.045*
Gender continuum scores		2.40	1, 84	0.015*
Block III				
Sex		1.90	1, 84	0.042*
Gender continuum scores		2.45	1, 84	0.017*
Sex × Gender continuum scores		2.66	1, 84	0.021*
	df	Pillai trace	F‐value	*p*‐value
Model 1	—	—	—	—
Model 2	1, 84	0.08	2.40	0.015*
Model 3	1, 84	0.14	2.66	0.021*

Abbreviations: df, degrees of freedom; FA, fractional anisotropy.

**TABLE 3:** Hierarchical linear regressions model outputs summarizing the significant effects of biological sex (sex) (block I) and gender continuum scores (gender) (block II) as well as their interactions (block III) on white matter fractional anisotropy (FA) for significant fronto‐limbic white matter tracts of interest, adjusting for age, highest level of education, and total intracranial volume.

	Beta	T‐value	P	Beta	T‐value	P	Beta	T‐value	P

**Left CST**									
Sex (male)	0.05	2.58	0.010*	0.06	2.88	0.004*	0.01	2.81	0.005*
Gender continuum scores	—	—	—	−0.04	−2.17	0.031*	−0.01	−2.28	0.022*
Sex × gender continuum scores	—	—	—	—	—	—	**0.05**	**2.88**	0.004*
*R* ^2^	0.145			0.166			0.177		
Δ*R* ^2^				0.021^a^			0.011^b^		
**Left SLF**									
Sex (male)	0.04	2.24	0.025*	0.04	2.31	0.021*	0.03	2.23	0.025*
Gender continuum scores	—	—	—	−0.03	−2.61	0.009*	−0.02	−2.88	0.008*
Sex × gender continuum scores	—	—	—	—	—	—	**0.04**	**3.00**	0.003**
*R* ^2^	0.135			0.145			0.155		
Δ*R* ^2^				0.010 ^a^			0.010^b^		
**Trunk of the corpus callosum**									
Sex (male)	0.13	2.75	0.006*	0.14	2.88	0.008*	0.15	2.58	0.010*
Gender continuum scores	—	—	—	−0.13	−2.61	0.009*	−0.15	−2.88	0.008*
Sex × gender continuum scores	—	—	—	—	—	—	**0.14**	**2.81**	0.005**
*R* ^2^	0.140			0.150			0.166		
Δ*R* ^2^				0.010 ^a^			0.016 ^b^		
**Right cingulum bundle**									
Sex (male)	0.11	1.21	0.224	0.11	1.22	0.221	0.09	1.19	0.234
Gender continuum scores	—	—	—	−0.13	−2.61	0.009*	−0.15	−2.75	0.012*
Sex × gender continuum scores	—	—	—	—	—	—	**0.13**	**2.88**	0.004*
*R* ^2^	0.138			0.148			0.160		
Δ*R* ^2^				0.010 ^a^			0.012^b^		
**Right IFOF**									
Sex (male)	0.06	1.53	0.125	0.11	1.51	0.131	0.09	1.24	0.212
Gender continuum scores	—	—	—	−0.16	−2.93	0.007*	−0.15	−2.51	0.013*
Sex × gender continuum scores	—	—	—	—	—	—	**0.13**	**2.85**	0.004**
*R* ^2^	0.139			0.150			0.165		
Δ*R* ^2^				0.011^a^			0.015^b^		
**Left UF**									
Sex (male)	0.05	1.43	0.155	0.07	1.45	0.149	0.07	1.48	0.141
Gender continuum scores	—	—	—	−0.16	−2.80	0.011**	−0.15	−2.75	0.012*
Sex × gender continuum scores	—	—	—	—	—	—	**0.13**	**2.81**	0.005**
*R* ^2^	0.144			0.152			0.168		
Δ*R* ^2^				0.008^a^			0.16^b^		

**indicates statistical significance following correction for multiple comparisons.

*indicates statistical significance at *p* < 0.05.

^a^addition of gender continuum score improved predictive capabilities of the model above and beyond that more biological sex alone.

^b^addition of sex × gender continuum score interaction term improved prediction capabilities above and beyond that for biological sex and gender scores as independent variables.

Abbreviations: CST, corticospinal tract; IFO,: inferior fronto‐occipital fasciculus; SLF, superior longitudinal fasciculus; UF, uncinate fasciculus.

### 3.5 Sex‐specific Associations Between Gender Continuum Scores and White Matter FA

Post‐hoc partial Pearson's correlations revealed that higher gender continuum scores were associated with lower white matter FA for the corpus callosum trunk, left CST, left SLF, right cingulum bundle, right IFOF, and left UF in males, adjusting for age and HLOE. There were no significant associations between gender continuum scores and white matter FA in females (Supplementary Table S4).

### 3.6 Sex‐specific Associations Between Masculine versus Feminine Gender Role Endorsement Scores and White Matter FA

Post‐hoc partial Pearson's correlations indicated that higher feminine gender role endorsement scores were associated with lower white matter FA for the trunk of the corpus callosum, left CST, left SLF, right cingulum bundle, right IFOF, and left UF in males, adjusting for age and HLOE. There were no significant associations between masculine gender role endorsement scores and white matter FA in males. No significant associations of white matter FA with masculinity or femininity scores in females were found (Table 4).

**TABLE 4:** Linear correlations between white matter fractional anisotropy (FA) for selected white matter tracts of interest that were significant in our multivariate linear regression model and masculine versus feminine gender role endorsement scores in males compared to females, adjusting for age, highest level of education, and total intracranial volume.

White matter FA	Masculine gender role endorsement scores	Feminine gender role endorsement scores
**Males**		
Corpus callosum trunk	0.11, 0.346	−0.25, 0.012*
Left CST	0.08, 0.826	−0.20, 0.043*
Left SLF	0.13, 0.819	−0.26, 0.026*
Right cingulum bundle	0.09, 0.442	−0.21, 0.027*
Right IFOF	0.05, 0.882	−0.30, 0.009*
Left UF	0.10, 0.411	−0.25, 0.031*
**Females**		
Corpus callosum trunk	0.23, 0.249	−0.05, 0.888
Left CST	0.18, 0.218	−0.06, 0.532
Left SLF	0.23, 0.433	−0.26, 0.353
Right cingulum bundle	0.25, 0.110	−0.04, 0.921
Right IFOF	0.09, 0.781	−0.30, 0.288
Left UF	0.12, 0.452	0.14, 0.115

*indicates statistical significance at *p* < 0.05.

Abbreviations: CST, corticospinal tract; FA, fractional anisotropy; IFOF, inferior fronto‐occipital fasciculus; SLF, superior longitudinal fasciculus; TIV, total intracranial volume.

## 4 Discussion

Our study revealed a novel relationship between gender role endorsement and white matter microstructural differences between males and females, suggesting sex‐specific patterns of association. We also replicated the well‐established finding that males have higher white matter FA than females (Hsu et al. 2008; Kanaan et al. 2012; Burke et al. 2017). The assessment of both sex and gender provided more nuanced insights into white matter microstructure compared to biological sex alone. To the best of our current knowledge, this is the first study that examined gender role endorsement as a continuous construct (rather than a binary measure) in relation to white matter microstructure in the general population.

We found that higher endorsement of feminine traits in males was associated with white matter patterns that resemble those observed in biological females. Gendered behavioral traits may therefore align with distinct white matter microstructure profiles. Earlier DTI studies have indeed shown that MtF transgender individuals have a pattern of white matter differentiation more typically observed in cisgender females, with lower FA in the corpus callosum trunk, CST, SLF, and cingulum bundle compared to cisgender men (Rametti et al. 2011a; Kranz et al. 2014; Burke et al. 2017). These findings provide a valuable point of comparison for observations noted in our sample: these parallels are, however, positioned as analogies, not direct equivalences.

Sex‐specific effects were also evident for the IFOF, which connects brain regions involved in self‐perception, facial recognition, and emotional regulation (Panesar et al. 2017). IFOF structural abnormalities can contribute to discrepancies between self‐perception and the physical body. Burke et al. (2017) reported “*sex‐reversed*” FA patterns for the IFOF in transgender individuals, while van Heesewijk et al. (2022) observed similar reductions in FA in transgender adolescents. Functional MRI studies corroborate these findings, having shown reduced parietal activation in MtF individuals relative to cisgender men (Carrillo et al. 2010; Schöning et al. 2010).

Lower white matter FA in fronto‐limbic tracts such as the cingulum bundle might reflect inter‐individual variation in emotion regulation, social cognition, and gendered behaviors. This is important, given how it suggests that gender identity, beyond biological sex, is related to structural differences in neural pathways supporting emotional and social processing. This highlights the need to consider psychological identity factors in brain–behavior research (Eliot 2025). Structural MRI studies have indeed revealed increased cortical thickness in the insula, anterior cingulate cortex, and orbitofrontal cortex in MtF individuals, aligning more closely with their gender identity than their natal sex (Luders et al. 2012; Zubiaurre‐Elorza et al. 2013). In addition to psychosocial influences, hormonal factors such as prenatal androgen exposure shape white matter development, interacting with environmental stressors to influence neural connectivity (Sheikh et al. 2014; O'Donovan et al. 2021). Taken together, these findings suggest that non‐normative gender expression in males may reflect differences in fronto‐limbic connectivity that could underscore inter‐individual variations in emotional processing and similar gendered behaviors.

Several limitations are acknowledged, including a moderate sample size, which limited generalizability of our findings, and a cross‐sectional design, which limited our ability to infer causality. In addition, combining data from two independent cohorts could have introduced potential differences in participant characteristics or scanning protocols. While we did not include statistical adjustment for the cohort, all imaging data underwent consistent quality control procedures. The BSRI is widely used but oversimplifies gender identity, as well as relying on self‐report and emphasizing gender roles rather than broader intersectional factors (e.g., independence, caregiver strain, risk‐taking, social support) (Rauch and Eliot 2022). It also does not formally assess transgender identities. Cis–trans distinctions were thus not applied in our sample. In South Africa, gender may reflect local socio‐cultural variations, underscoring the need for a culturally sensitive interpretation of gender–brain associations in a general population setting (Gibbs et al. 2014). It is salient to further emphasize that most BSRI validation studies were conducted in Western populations. The constructs measured using this scale might thus not fully align with the lived experiences of gender roles in the South African setting.

That being said, the present study also has notable strengths. Our approach differs from that of most prior DTI studies that examined the associations of gender identity with white matter structure. We operationalized gender along a continuous spectrum of role endorsement, rather than as a categorical variable. This allowed us to examine subtle inter‐individual variation beyond cis–trans distinctions. We further used a validated instrument to assess masculine and feminine gender role endorsement as discrete constructs. A major strength further lies in the integration of biological sex with gender role endorsement as a continuous construct, indicating a shift that extends beyond binary frameworks used in prior DTI studies.

## 5 Conclusions

In summary, we identified sex‐ and construct‐specific associations between gender role endorsement and white matter microstructure in healthy South African adults. Integrating sex and gender constructs in neuroimaging enhances understanding of brain structure and health disparities. Longitudinal studies are needed to determine whether gender‐related FA differences reflect developmental processes influenced by environmental risk exposures (Eliot 2025). Future neuroimaging studies would do well to incorporate multidimensional or qualitative assessments of gender identity to provide a nuanced understanding of gendered experience in relation to brain structure. This can help clarify the underlying neural correlates of non‐binary gender behaviors within broader bio‐psycho‐social frameworks as well as specific cultural milieus.

## Author Contributions

Hilmar Klaus Luckhoff conceptualized the study, curated the data, conducted the formal analysis and investigation of the data, formulated and applied the methodology, and prepared the initial draft of the manuscript. Retha Smit assisted with data curation and helped coordinate the study. Robin Emsley assisted with conceptualization of the study, acquired necessary funding, and supervised the study. Laila Asmal assisted with funding acquisition, provided the necessary study resources, and helped supervise the research. Retha Smit, Lebogang Phahladira, Sanja Kilian, Robin Emsley, and Laila Asmal all helped review and edit the final draft of the manuscript.

## Ethics Statement

Ethics approval for the present study was obtained from the Health Research Ethics Committee at the Faculty of Medicine and Health Sciences of Stellenbosch University.

## Consent

All participants provided written, informed consent for research participation and secondary analyses of data.

## Funding

This study was funded by New Partnership for Africa's Development (NEPAD) grant, through the Department of Science and Technology of South Africa, the Medical Research Council of South Africa “SHARED ROOTS” Flagship Project Grant no. MRCRFA‐IFSP‐01‐2013 (Grant holder S Seedat), and an unrestricted grant from Lundbeck International. The content of this publication is solely the responsibility of the authors and does not necessarily represent the official views of the SAMRC. The funding sources had no role in the design of the study, nor during its execution, analyses, interpretation of results, and drafting of the manuscript.

## Conflicts of Interest

The authors declare no conflicts of interest.

## Supporting information




**Supplementary Tables**: brb371340‐sup‐0001‐Tables.docx

## Data Availability

The data used in our research are available upon reasonable request to the corresponding author.
