# Supplementary material for: Sex versus Gender Role Endorsement Associations With White Matter Microstructure in South African Adults: A Diffusion Tensor Imaging Study
Source: Brain Behav. 2026 Mar 31;16(4):e71340. doi: 10.1002/brb3.71340 (PMC13112026; doi:10.1002/brb3.71340)
Supplement: Supplementary file 1 — Supplementary Tables: brb371340‐sup‐0001‐Tables.docx [file BRB3-16-e71340-s001.docx]

**SUPPLEMENTAL MATERIALS**

Supplemental Table S1. Sample characteristics. Data are presented as the mean along with standard deviation (SD).

|  | **Male (n = 46)** | **Female (n = 42)** | **T-statistic** | **P-value** |
| --- | --- | --- | --- | --- |
| Age, years | 27.7 (11.3) | 30.9 (10.2) | -1.39 | 0.17 |
| HLOE, years | 10.7 (1.3) | 11.0 (1.6) | -1.01 | 0.32 |
| Gender continuum scores | -0.18 (1.01) | 0.16 (0.8) | -1.67 | 0.082 |
| BSRI masculine GRE scores | 5.11 (0.75) | 5.06 (0.59) | 0.36 | 0.72 |
| BSRI feminine GRE scores | 4.98 (0.68) | 5.22 (0.59) | -1.78 | 0.077 |

BSRI: Bem Sex Role Inventory; GRE: gender role endorsement; HLOE: highest level of education

Supplemental Table S2. Hierarchical regression model outputs summarizing the significant effects of biological sex (block I) and gender continuum scores (block II) as well as their interactions (block III) on global white matter fractional anisotropy (FA) in our sample, adjusting for age, highest level of education, and total intracranial volume.

|  | Beta | T-value | P | Beta | T-value | P | Beta | T-value | P |
| --- | --- | --- | --- | --- | --- | --- | --- | --- | --- |
| Global white matter FA | | | | | | | | | |
| Sex (male) | 0.01 | 0.83 | 0.41 | 0.01 | 0.29 | 0.77 | 0.01 | 0.51 | 0.85 |
| Gender | - | - | - | **-0.13** | **-2.43** | **0.019*** | **-0.14** | **-2.06** | **0.039*** |
| Sex × gender | - | - | - | - | - | - | **0.26** | **2.00** | **0.047*** |
| R^2^ | 0.110 | | | 0.136 | | | 0.146 | | |
| ΔR^2^ |  | | | 0.026^a^ | | | 0.010^b^ | | |

* indicates statistical significance at p <0.05

a: addition of gender continuum scores improved predictive capabilities of the model above and beyond that more biological sex alone

b: addition of sex × gender continuum score interaction term improved prediction capabilities of the model above and beyond that for biological sex and gender scores as independent variables

Supplemental Table S3. Hierarchical regressions outputs summarizing the significant effects of biological sex (sex) (block I) and gender continuum scores (gender) (block II) as well as their interactions (block III) on white matter fractional anisotropy (FA) for non-significant fronto-limbic white matter tracts of interest, adjusting for age, highest level of education, and total intracranial volume.

|  | **Beta** | **T-value** | **P** | **Beta** | **T-value** | **P** | **Beta** | **T-value** | **P** |
| --- | --- | --- | --- | --- | --- | --- | --- | --- | --- |
|  | | | | | | | | | |
| **Genu of corpus callosum** | | | | | | | | | |
| Sex (male) | 0.01 | 0.16 | 0.876 | 0.01 | 0.23 | 0.882 | 0.01 | 1.55 | 0.895 |
| Gender continuum scores | - | - | - | -0.01 | -0.36 | 0.722 | -0.01 | -0.43 | 0.831 |
| Sex × gender continuum scores | - | - | - | - | - | - | 0.01 | 0.83 | 0.916 |
| R^2^ | 0.082 | | | 0.088 | | | 0.093 | | |
| ΔR^2^ |  | | | 0.006 | | | 0.005^b^ | | |
| **Fornix** | | | | | | | | | |
| Sex (male) | -0.01 | -0.82 | 0.418 | -0.01 | -0.71 | 0.481 | -0.01 | -0.79 | 0.436 |
| Gender continuum scores | - | - | - | -0.01 | -0.39 | 0.696 | -0.01 | -0.46 | 0.646 |
| Sex × gender continuum scores | - | - | - | - | - | - | 0.01 | 0.36 | 0.724 |
| R^2^ | 0.093 | | | 0.096 | | | 0.100 | | |
| ΔR^2^ |  | | | 0.003 | | | 0.004 | | |
| **Splenium of corpus callous** | | | | | | | | | |
| Sex (male) | -0.02 | -0.23 | 0.821 | -0.02 | -0.26 | 0.792 | -0.02 | -0.29 | 0.810 |
| Gender continuum scores | - | - | - | 0.01 | 0.22 | 0.912 | 0.01 | 0.21 | 0.944 |
| Sex × gender continuum scores | - | - | - | - | - | - | -0.01 | -0.33 | 0.341 |
| R^2^ | 0.089 | | | 0.094 | | | 0.097 | | |
| ΔR^2^ |  | | | 0.005 | | | 0.003 | | |
| **Right CST** | | | | | | | | | |
| Sex (male) | -0.01 | -0.13 | 0.925 | -0.01 | -0.15 | 0.889 | -0.01 | -0.14 | 0.858 |
| Gender continuum scores | - | - | - | -0.01 | -0.35 | 0.782 | -0.01 | -0.38 | 0.723 |
| Sex × gender continuum scores | - | - | - | - | - | - | 0.01 | 0.40 | 0.681 |
| R^2^ | 0.088 | | | 0.092 | | | 0.096 | | |
| ΔR^2^ |  | | | 0.004 | | | 0.004 | | |
| **Right SLF** | | | | | | | | | |
| Sex (male) | 0.01 | 0.24 | 0.725 | 0.01 | 0.22 | 0.692 | 0.01 | 0.26 | 0.612 |
| Gender continuum scores | - | - | - | -0.01 | -0.43 | 0.667 | -0.01 | -0.46 | 0.353 |
| Sex × gender continuum scores | - | - | - | - | - | - | 0.01 | 0.66 | 0.252 |
| R^2^ | 0.080 | | | 0.085 | | | 0.089 | | |
| ΔR^2^ |  | | | 0.005 | | | 0.004 | | |
| **Left cingulum bundle** | | | | | | | | | |
| Sex (male) | 0.01 | 0.39 | 0.801 | 0.01 | 0.35 | 0.788 | 0.01 | 0.45 | 0.721 |
| Gender continuum scores | - | - | - | 0.01 | 0.52 | 0.607 | -0.01 | -0.66 | 0.513 |
| Sex × gender continuum scores | - | - | - | - | - | - | 0.01 | 0.85 | 0.388 |
| R^2^ | 0.079 | | | 0.084 | | | 0.088 | | |
| ΔR^2^ |  | | | 0.005 | | | 0.004 | | |
| **Left IFOF** | | | | | | | | | |
| Sex (male) | -0.01 | -0.12 | 0.907 | 0.01 | 0.21 | 0.788 | 0.01 | 0.10 | 0.924 |
| Gender continuum scores | - | - | - | -0.01 | -0.84 | 0.133 | -0.01 | -0.72 | 0.525 |
| Sex × gender continuum scores | - | - | - | - | - | - | 0.01 | 0.48 | 0.818 |
| R^2^ | 0.070 | | | 0.075 | | | 0.079 | | |
| ΔR^2^ |  | | | 0.005 | | | 0.004 | | |
| **Right UF** | | | | | | | | | |
| Sex (male) | -0.01 | -0.33 | 0.620 | -0.01 | -0.28 | 0.788 | 0.01 | -0.50 | 0.620 |
| Gender continuum scores | - | - | - | -0.01 | -0.76 | 0.177 | -0.01 | -0.69 | 0.495 |
| Sex × gender continuum scores | - | - | - | - | - | - | 0.01 | 0.55 | 0.580 |
| R^2^ | 0.080 | | | 0.083 | | | 0.087 | | |
| ΔR^2^ |  | | | 0.003 | | | 0.004 | | |

CST: corticospinal tract; IFOF: inferior fronto-occipital fasciculus; SLF: superior longitudinal fasciculus; UF: uncinate fasciculus

Supplemental Table S4. Linear correlations between gender continuum scores and white matter fractional anisotropy (FA) for selected white matter tracts of interest that were significant in our multivariate linear regression model in males compared to females, adjusting for age, highest level of education, and total intracranial volume.

| **White matter FA** | **Males** | **Females** |
| --- | --- | --- |
|  | Gender continuum scores  Rho, p-value | Gender continuum scores  Rho, p-value |
| Corpus callosum trunk | **-0.23, 0.009*** | 0.05, 0.454 |
| Left CST | **-0.22, 0.011*** | -0.13, 0.221 |
| Left SLF | **-0.30, 0.015*** | -0.19, 0.154 |
| Right cingulum bundle | **-0.29, 0.022*** | -0.14, 0.332 |
| Right IFOF | **-0.32, 0.004*** | -0.29, 0.166 |
| Left UF | **-0.30, 0.031*** | 0.25, 0.255 |

CST: corticospinal tract; IFOF: inferior fronto-occipital fasciculus; SLF: superior longitudinal fasciculus; UF: uncinate fasciculus. * indicates statistical significance at p <0.05.
